# Abdominal surgical trajectories associated with failure to rescue. A nationwide analysis

**DOI:** 10.1093/intqhc/mzac084

**Published:** 2022-10-26

**Authors:** Katrine Skyrud, Jon Helgeland, Anne Karin Lindahl, Knut Magne Augestad

**Affiliations:** Cluster for Health Services Research, Norwegian Institute of Public Health, PO Box 222 Skøyen, Oslo N-0213, Norway; Cluster for Health Services Research, Norwegian Institute of Public Health, PO Box 222 Skøyen, Oslo N-0213, Norway; Division of Surgery, Akershus University Hospital, Lørenskog, Oslo 1478, Norway; Institute of Cliniacal Medicine, University of Oslo, Problemveien 7, Oslo 0315, Norway; Division of Surgery, Akershus University Hospital, Lørenskog, Oslo 1478, Norway; Institute of Cliniacal Medicine, University of Oslo, Problemveien 7, Oslo 0315, Norway; Department of Quality and Research, University Hospital North Norway, Sykehusvegen 38, Tromsø 9019, Norway; Department of Surgery, Helgelandssykehuset, Prestmarkveien 1, Sandnessjøen 8800, Norway

**Keywords:** abdominal surgery, failure to rescue, complications, 30-day mortality

## Abstract

**Objective:**

The ability to detect and treat complications of surgery early is essential for optimal patient outcomes. The failure-to-rescue (FTR) rate is defined as the death rate among patients who develop at least one complication after the surgical procedure and may be used to monitor a hospital’s quality of surgical care. The aim of this observational study was to explore FTR in Norway and to see if we could identify surgical trajectories associated with high FTR.

**Method:**

Data on all abdominal surgeries in Norwegian hospitals from 2011 to 2017 were obtained from the Norwegian Patient Registry and linked with the National Population Register. Surgical and other postoperative complication rates and FTR within 30 days (deaths occurring in and out of the hospital) were assessed. We identified surgical trajectories (type of procedures—type of complication—dead/alive at 30 days after operation) associated with the highest volume of deaths (high volume of FTR [FTR-V]) and highest risk of death after a postoperative complication.

**Results:**

Of the total 626 052 primary abdominal procedures, 224 871 (35.8%) had at least one complication, which includes 83 037 patients. The most common postoperative complications were sepsis (*N* = 14 331) and respiratory failure (*N* = 7970). The high-volume trajectories (FTR-V) were endoscopic retrograde cholangiopancreatography—sepsis—death (*N* = 294, 13.8%); open colon resections—sepsis—death (*N* = 279, 28.1%) and procedures with stoma formation—sepsis—death (*N* = 272, 27%). Similarly, patients operated with embolectomy of the visceral arteries and experiencing postoperative sepsis were associated with an extremely high risk of 30-day FTR of 81.5%. In general, an FTR patient had a higher mean age, an increased rate of emergency surgery and more comorbidity. Hospital size was not associated with FTR.

**Conclusion:**

At a national level, there exist high-volume and high-risk surgical trajectories associated with FTR. These trajectories represent major targets for quality improvement initiatives.

## Introduction

Continuous quality improvement in high-quality surgical care is essential, and quality measures in surgery, especially in Norway, are scarce. Postoperative complications are common and are estimated to occur in approximately 18–23% of patients [1, 2]. To explore postoperative complications after surgery, the rate of failure to rescue (FTR) is a valid marker of surgical complication management. Still, it has not yet been assessed in Norway. The FTR rate is defined as the death rate among surgical patients who develop at least one complication during the hospital stay. It reflects the ability of a hospital to detect and provide appropriate care to surgical patients after a complication that may be preventable. A high FTR rate may identify high-risk settings, whereas a high FTR volume indicates a high overall disease burden.

According to the international literature, FTR is multifactorial and institutional, and patient factors are associated with FTR [3]. Studies investigating the effect of hospital characteristics on FTR following major surgery have found that the incidences of both perioperative complications and FTR vary depending on the type of procedure, complication, and kind of hospital [4–8]. To reduce the FTR rate, temporal progression from the index surgical procedure and the potential complications associated with FTR must be explored [3].

With these goals in mind, this study’s objective was to analyze the FTR risk and volume in a complete national cohort of abdominal surgical patients. We wanted to identify the abdominal surgical trajectories most commonly associated with FTR. Note that we do not intend to compare hospitals or study the cause of FTR; therefore, no risk adjustment was performed in the analysis when determining surgical trajectories. We hypothesized that specific ‘high-volume’ surgical trajectories would have a higher FTR volume than others. Ultimately, this knowledge may be utilized by health-care workers and policymakers to target quality improvement programs for surgical trajectories with a high volume of FTR (FTR-V). Targeting these ‘high-volume’ surgical trajectories would significantly decrease the number of deaths caused by FTR.

The main objectives of this article are to

- identify surgical trajectories with FTR-V and
- for trajectories with FTR-V, identify important patient characteristics associated with FTR.

## Methods

### Patient population and data source

From the Norwegian Patient Registry (NPR), we obtained patient administrative data from all publicly financed Norwegian hospitals for the period 2011–7. The dataset contained type of admission (acute or elective), primary and secondary diagnosis codes according to the Norwegian version of International Classification of Diseases 10th Revision (ICD-10), surgical and medical procedures, age, sex, date and time of ward admission and discharge, and procedures for all department stays. Surgical procedures were coded according to the Norwegian version of the Nordic Medico-Statistical Committee (NOMESCO) Classification of Surgical Procedures (NCSP-N) [9]. The NCSP does not identify the intent or type of operation, and it only covers single procedures.

All permanent residents of Norway have a personal identification number (PIN). Hospital data were linked with the National Population Register to provide the date of death (where applicable) using the encrypted PIN.

### Surgical procedures and postoperative complications

The initial data set of procedures included all abdominal surgical procedures (Supplement Table S6). The procedures were classified as gastrointestinal, gynecological, urological or vascular procedures, including elective or emergency procedures. Procedures with a missing PIN, admission type, or vital status or a recorded date of death more than 24 hours before department admission were excluded. Procedures on patients aged 18 years or older whose hospital episodes spanned at least from one date to the next were included (e.g. day cases were excluded). In the case of a missing procedure start or end time of day, these were imputed as 0900 and 1500, respectively. The procedure codes included in this sample are listed according to the surgical procedure in the Supplementary Table S6. Several procedures may be linked to one ‘operation’ if the time intervals overlap. In addition, similar procedures were grouped together in the statistical analyses, i.e. for instance, they consisted of stoma formation of the procedures ‘JJF00 Catheter enterostomy’, ‘JFF10 Loop enterostomy’, ‘JFF11 Laparoscopic loop enterostomy’, ‘JFF13 Terminal enterostomy’, ‘JFF20 Caecostomy’, ‘JFF23 Transversostomy’, ‘JFF26 Sigmoideostomy’ and ‘JFF27 Laparoscopic sigmoidostomy’ (Supplementary Table S6).

Postoperative complications were defined from diagnosis codes as shown in the Supplementary Table S5 and were identified after each ‘operation’. Our final study population included all surgical procedures that were followed by at least one reported postoperative complication. Hence, if a patient had more than one complication, the patient was counted for each complication they suffered from. Similarly, if a patient had more than one surgery during the study period, we include all of the admissions. Also see the flowchart in Supplementary Figure S1 for a graphical display of the inclusion and exclusion of the study population.

### Outcomes

The primary outcome was FTR 30-day mortality (30-day FTR), defined as death within 30 days of a surgical patient who developed at least one complication during the hospital stay, both as a rate and as a volume. Originally, FTR was defined as the death during hospitalization of surgical patients who developed at least one complication during the hospital stay. We used a revised definition of FTR (death (in-or-out of the hospital) within 30 days after operation instead of death during hospitalization), as 30-day mortality avoids the bias of in-hospital mortality due to variations in length of stay [10]. The comorbidities were those included in the Quan *et al*. version of the Charlson comorbidity index, determined from previous admissions 3 years prior to but not including the current episode of care [11, 12].

### Identification of surgical trajectories associated with FTR

Exploration of the disease trajectory was used as a framework for studying patterns and visualizing the temporal disease patterns associated with FTR [13]. We defined the surgical trajectories as type of procedures—type of complication—dead/alive at 30 days after operation. To visualize, we used a Sankey diagram, which is used to depict a flow from one set of values to another. A Sankey diagram is a type of flow diagram in which the arrow width is proportional to the flow volume/rate. To identify specific surgical trajectories associated with high 30-day FTR, we identified the combination of procedure and one complication resulting in:

- the highest volume of 30-day FTR (FTR-V), i.e. number of deaths 30 days after surgery and
- the highest risk of 30-day FTR (high risk of FTR [FTR-R]), i.e. number of deaths 30 days after surgery divided by the number of procedures with that specific combination of procedure and postoperative complication.

One surgical procedure could have more than one postoperative complication. In that case, FTR-V and FTR-R were calculated for all combinations, hence including all complications. Each procedure type was defined by surgical specialty. The analysis was done for all hospitals as well as separately for large (>2800 operations per year), medium (570–2800 per year) and small (<570 per year) hospitals. To identify the primary surgical procedure, we excluded the general procedure codes marked with italics in Supplementary Table S6. In addition, surgical trajectories with fewer than 10 procedures were excluded from the analysis.

Surgical trajectories with the highest volume of 30-day FTR (FTR-V) were characterized as ‘high-volume’ surgical trajectories, while those with the highest risk of 30-day FTR (FTR-R) were characterized as ‘high-risk’ surgical trajectories (purely descriptive).

As a next step, after identifying the most important ‘high-volume’ trajectories (FTR-V), we performed a sub-analysis for identifying important patient characteristics associated with FTR. A multivariable logistic regression for each combination of surgical procedure and complication was fitted with death within 30 days as the outcome and age, sex, type of admission, Charlson index and hospital size as explanatory variables. The *P*-value for each of the explanatory variables (from the logistic regression) was adjusted for multiple testing by Holm’s method.

All statistical analyses were performed with R, version 3.5.1, and we visualized the surgical pathway (the Sankey diagram) using the R package ggalluvial (https://cran.r-project.org/web/packages/ggalluvial/vignettes/ggalluvial.html).

### Ethical approval

The study was approved by the Norwegian Directorate of Health and the Norwegian Data Protection Authority, and it is consistent with the Strengthening the Reporting of Observational Studies in Epidemiology statement [14].

## Results

A total of 626 052 primary abdominal procedures (out of 407 113 operations) were performed in the period 2011–7 (Table 1). The number of procedures with at least one complication was 224 871, resulting in an overall complication rate of 35.8%, including 83 037 patients. Most surgical procedures were elective, except 44.0% of gastrointestinal surgeries were elective procedures. The average age was 66.5 years, and males had a higher complication rate than females. The most common comorbidities were malignancy (including lymphoma and leukemia, excluding malignant neoplasm of skin) (24.8%), followed by chronic pulmonary disease (9.8%) and renal disease (9.8%), with some variation across the surgical specialties. The highest complication rate (56.3%) was seen for vascular procedures, followed by gastrointestinal surgery (46.3%), urological surgery (30.0%) and gynecological surgery (10.3%). The highest 30-day FTR rate was observed in gastrointestinal surgery (10.0%), followed by vascular surgery (9.8%) (Supplementary Table S1). The lowest 30-day FTR rate was observed for gynecological surgery (1.0%) and urological surgery (2.5%).

**Table 1:** Descriptive statistics of the population by surgical specialty

	All abdominal surgery	Vascular	Gastrointestinal	Gynecology	Urology
Total procedures	626 052	27 228	291 009	110 684	146 686
Procedures with at least one complication (%)	224 871 (35.8)	15 338 (56.3)	135 375 (46.5)	11 369 (10.3)	44 139 (30.1)
Age (mean)	66.5	72.5	66.0	59.1	68.9
Elective admissions (%)	54.9	52.8	44.0	82.5	73.1
Male (%)	52.7	62.0	53.8	0.2	69.9
Charlson index (mean)	1.4	1.0	1.4	0.5	1.4
Most common comorbidities (%)					
Any malignancy^a^	24.8	7.6	25.1	7.6	30.3
Chronic pulmonary disease	9.8	15.5	9.7	5.7	10.0
Renal disease	8.3	12.0	6.8	2.2	12.7
Congestive heart failure	7.3	13.4	6.8	3.6	8.2
Metastatic disease	6.5	1.8	7.9	1.5	5.2

a Any malignancy, including lymphoma and leukemia, except malignant neoplasm of the skin.

Overall, cardiac arrest was the complication with the highest FTR-R (45.8%), while sepsis resulted in the highest FTR-V (2554 number of deaths) (Supplementary Table S1). For patients undergoing gastrointestinal surgery with subsequent cardiac arrest, 47.0% died within 30 days, while the highest number of deaths (*N* = 2029) occurred after postoperative sepsis (Supplementary Table S1). Similar to other surgical specialties, gynecological and vascular surgeries also had the highest FTR-R caused by acute ischemia of the bowel and cardiac arrest.

Overall, open colon resections and endoscopic retrograde cholangiopancreatography (ERCP) had the highest number of failures to rescue within 30 days, with 1080 and 982 deaths, respectively (Supplementary Table S2). On the other hand, embolectomy of the visceral arteries was the procedure associated with the highest FTR-R (35.2%), followed by colon stenting (19.8%).

### Surgical trajectories associated with FTR-V

**Figure 1:**
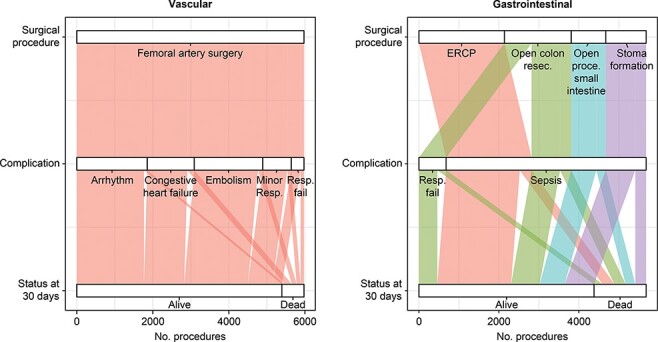
Vascular and gastrointestinal surgical trajectories associated with FTR-V. The temporal nodes (read from top to bottom) are the primary surgical procedure, postoperative complication and 30-day failure-to-rescue rate. For the high-risk surgical procedures and subsequent FTR, see the Supplementary Table S4. ERCP: endoscopic retrograde cholangiography.

Within vascular surgeries, the highest number of deaths occurred with femoral artery surgery, followed by congestive heart failure (179) or embolism (125) (Figure 1, Table 2). These results were largely independent of the size of hospitals (size of hospitals was not significant as an explanatory variable for FTR) (Table 3), although few of the vascular procedures were performed at small hospitals (Table 2). Furthermore, the surgical trajectory, femoral artery surgery—congestive heart failure—death, indicated that risk of FTR is higher if the patients were old, male or had comorbidity (especially chronic pulmonary disease, cancer or metastatic disease) (Table 3).

**Table 2:** Surgical trajectories associated with FTR-V

			All hospitals		Large hospitals	Medium hospitals	Small hospitals
Specialty	Surgical procedure	Complication	FTR/procedures	30-Day FTR-V	30-Day FTR-V	30-Day FTR-V	30-Day FTR-V
Vascular	Femoral artery	Congestive heart failure	179/1236	14.5	13.6	15.4	
	Femoral artery	Embolism	125/1802	7.0	7.9	4.4	22.2
	Femoral artery	Minor respiratory complications^a^	103/749	13.8	14.7	14.0	
	Femoral artery	Respiratory failure	89/336	26.5	32.2	23.5	
	Femoral artery	Arrhythmia	85/1853	4.6	5.4	2.8	
GI surgery	ERCP	Sepsis	294/2138	13.8	12.8	15.1	10.6
	Open colon resections	Sepsis	279/993	28.1	30.5	20.8	31.0
	Stoma formation	Sepsis	272/1008	27.0	26.8	26.0	22.5
	Open small bowel resections	Sepsis	239/865	27.6	30.5	22.7	23.6
	Open colon resections	Respiratory failure	219/679	32.3	32.6	31.5	39.1
Urology	Ureter stents	Sepsis	108/1570	6.9	8.3	4.6	6.4
	Ureter stents	Acute kidney failure	64/1158	5.5	5.1	5.8	7.7
	Ureter stents	Minor respiratory complications ^a^	55/1467	3.7	3.6	4.0	7.1
	Vesical	Sepsis	52/354	14.7	17.8	11.3	19.4
	Ureter stents	Urinary infection	44/3321	1.3	1.4	0.8	2.0
Gynecology	Ovarian	Respiratory failure	17/98	17.3	13.3	19.0	
	Uterus	Respiratory failure	15/117	12.8	6.5	18.3	16.7
	Ovarian	Sepsis	14/152	9.2	10.9	8.0	8.3
	Uterus	Minor respiratory complications^a^	13/1036	1.3	1.0	1.8	0.9
	Uterus	Sepsis	13/183	7.1	4.6	7.7	6.3

a Minor respiratory complications include asthma, pleura effusion and dyspnea.

Femoral artery: femoral artery surgery.

**Table 3:** Patient characteristics for trajectories with the highest volume of FTR (FTR-V)

	ERCP and sepsis			Open colon resections and sepsis			Femoral artery surgery and congestive heart failure			Femoral artery surgery and embolism		
	No FTR	FTR	*P*	No FTR	FTR	*P*	No FTR	FTR	*P*	No FTR	FTR	*P*
Number of procedures	1844	294		714	279		1057	179		1677	125	
Age (mean)	73.6	75.2	0.001	66.6	73.6	<0.001	76.7	79.5	0.001	73.1	82.9	n.s
Elective admissions (%)	9.0	14.6	0.002	30.0	20.1	0.001	56.8	30.2	0.001	27.4	7.2	0.05
Male (%)	56.5	64.3	0.001	46.2	49.1	n.s	59.2	60.3	0.001	54.1	49.6	n.s
Charlson, index (mean)	1.4	2.9	<0.001	1.2	1.5	n.s	2.3	1.8	0.001	0.9	1.8	<0.001
Hospital size												
Large	1064	156	n.s	388	170	n.s	719	113	n.s	1044	89	n.s
Medium	629	112		247	65		291	53		480	22	
Small	110	13		113	78					14	4	
Most common comorbidities (%)												
None	46.8	24.8		52.0	45.2		14.9	25.8		55.3	37.7	
Any malignancy^a^	14.1	26.8		19.4	13.6		3.9	5.2		7.9	16.2	
Chronic pulmonary disease	7.9	6.7		7.1	10.8		15.5	13.8		11.1	10.8	
Renal disease	7.7	8.5		4.7	6.8		14.9	16.4		5.9	9.0	
Congestive heart failure	17.	8.9		4.4	9.7		37.2	29.1		6.9	7.2	
Metastatic disease	5.8	15.8		5.7	6.5		0.3	1.9		2.9	8.4	

a Any malignancy, including lymphoma and leukemia, except malignant neoplasm of the skin.

In total, if we considered all hospitals together, the highest numbers of deaths occurred with the frequent gastrointestinal procedures ERCP, stoma formation and open colon resection, followed by a complication of sepsis (294, 279, 272) (Figure 1, Table 2). Furthermore, the trajectory ERCP—sepsis—death indicated that risk of FTR is higher if the patients were old, male or had comorbidity (especially cancer or metastatic disease) (Table 3). However, for the pathway of open colon resections and sepsis, only age was significant as explanatory variable for FTR. In addition, the size of hospitals was not significant as an explanatory variable for FTR.

**Figure 2:**
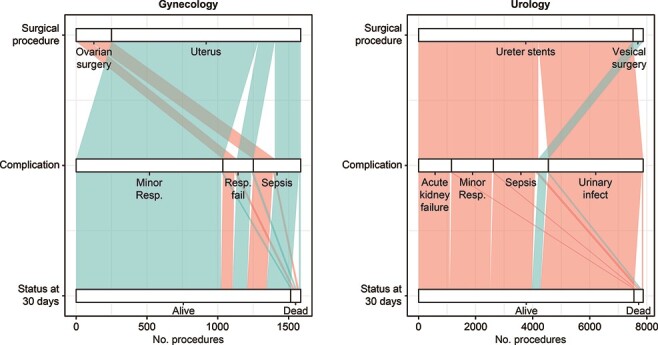
Gynecological and urological surgical trajectories associated with FTR-V. The temporal nodes (following the flow from top to bottom) are the primary surgical procedure, postoperative complication and 30-day FTR rate. For the high-risk surgical procedures and subsequent FTR, see the supplement Table S4. Uterus: hysterectomies and other surgeries on uterus.

Within urological surgeries, the highest number of deaths occurred with ureter stent surgery, followed by sepsis or acute kidney failure (108 and 64, respectively) (Figure 2, Table 2). The exception was ureter stents followed by acute kidney failure, where the death occurred only in 9 out of 277 elective procedures (3.2%) compared to 55 out of 881 acute procedures (6.2%). Age was a significant factor for explaining FTR in the trajectories of ureter stent surgery, followed by either sepsis or acute kidney failure, while comorbidity was only significant for the trajectory of ureter stents and sepsis (Supplementary Table S3). The size of hospitals was significant in neither of the trajectories.

Within gynecological surgeries, the highest number of deaths occurred with ovarian or uterus surgery, followed by respiratory failure, with 17 and 15 deaths, respectively (Figure 2, Table 2).

In addition, no patient characteristics or size of the hospital was significantly different between FTR versus no FTR (Supplementary Table S3).

### Surgical trajectories associated with FTR-R

Patients who were operated with embolectomy of the visceral arteries and experiencing postoperative sepsis were associated with an extremely high risk of 30-day FTR of 81.5% (Supplementary Table S4). Besides, we observed a markedly high FTR-R for patients operated for liver surgery with postoperative cardiac arrest (75.0%) (Supplementary Table S4). Although lower FTR-R in patients operated for urology surgery, patients undergoing vesical surgery and postoperative cardiac arrest were associated with an FTR-R of 65.6% (Supplementary Table S4). Furthermore, to be noted is the high FTR-R after ovarian surgery and postoperative acute ischemia of the bowel of 38.9% (Supplementary Table S4).

## Discussion

### Statement of principal findings

This study has shown that distinct surgical trajectories are associated with 30-day FTR in a large national sample of patients undergoing abdominal surgery. Patients experiencing 30-day FTR often had a high rate of comorbidities; cancer, heart disease and Chronic obstructive pulmonary disease were the most common. Gastrointestinal surgery had the highest number of deceased patients and was most often associated with FTR, followed by vascular surgery and urology. The complications that were most frequently followed by death within 30 days were cardiac arrest, acute bowel ischemia, respiratory failure and sepsis. Importantly, we identified specific gastrointestinal and vascular procedures that are high-volume procedures positively associated with congestive heart failure, sepsis, respiratory failure and subsequent 30-day FTR.

### Interpretation within the context of the wider literature

If viewed in isolation, postoperative complications and mortality are suboptimal measurements of quality, as they are more associated with patient factors (i.e. frailty) and less with hospital characteristics and delivery of care [15]. To reduce the FTR rate, the temporal progression from the index surgical procedure and the potential complications associated with FTR must be explored [3]. In a recent study, Giuliani *et al.* identified three major patterns leading to postoperative mortality [16]. They concluded that mortality after pancreatoduodenectomy occurs through three distinct pathways relying on different patient characteristics, in line with our study. They also argue that this is an additional tool to identify the best targets for quality improvement. In addition, Sujan *et al.* concluded that Functional Resonance Analyses Method can be used to study surgical work systems to identify quality improvement recommendations [17]. Future efforts to reduce FTR variations between units may benefit from using this framework for quality improvement [18].

### Implications for policy, practice and research

The study has several important findings. To our knowledge, this study is the first to explore specific ‘high-volume’ surgical trajectories associated with 30-day FTR. National quality improvement initiatives should target these trajectories. The main objective of this article is to identify surgical trajectories with a high volume of FTR, and thereby it is not relevant to adjust for factors affecting 30-day FTR. Analysis controlling for patient characteristics would result in a list of pathways that are in some sense unexpected given the patient’s health status. We believe that these pathways would only account for a minor fraction of the total FTRs and therefore be of little relevance for planning quality improvement measures.

Second, some surgical procedures had an alarmingly high FTR rate. Of note are embolectomies and surgeries on the visceral arteries, as these procedures were associated with sepsis and subsequent FTR. We found that 81.5% of the patients (see Supplementary table S4) died within 30 days. It might be questioned whether these procedures are performed too often. We encourage surgeons, health-care workers and policymakers to recognize these high-risk FTR trajectories and to initiate a continuous discussion about the value of the procedures, i.e. their outcomes and benefits [19, 20].

Third, we believe it is essential to differentiate between preventable and unpreventable causes of FTR. FTR caused by complications such as cardiac arrest and circulatory collapse may be part of terminal illness, and it might be questioned whether this should even be classified as FTR. Of note is that many of these procedures are related to palliative cancer surgery, performed in the terminal stages of cancer, where patient death is expected within a short time frame [21].

In our opinion, preventable complications must be identified and targeted in quality improvement initiatives by health-care workers and decision-makers. This view has been echoed by others, especially with the emergence of public reporting of outcomes and an increased focus on pay-for-performance models [15, 18].

### Strengths and limitations

Our study’s important strength is the use of complete data, including every patient who underwent abdominal surgery at all Norwegian hospitals over 7 years. It is mandatory for hospitals to report any type of contact, and the registry is complete and does not suffer from population bias, i.e. demographic bias or medical specialty bias [22]. We believe that the uniqueness of our analyses is in their identification of the significant FTR trajectories at a national level. Another valuable strength is that we explored the surgical trajectories in two ways: (i) identified the trajectories associated with the highest risk of FTR and (ii) the trajectories leading to the highest number of deaths. It is essential to be aware of both aspects; a high FTR rate may determine high-risk settings, whereas a high FTR volume indicates a high overall disease burden. The largest reduction in the total FTR rate would be achieved if improvement were targeted on trajectories with an increased risk of FTR and high numbers of deaths.

There are several limitations. First, although the data are based on a large and complete national inpatient sample, the results must be interpreted with care. Some surgical procedures have low volume and a low frequency of complications associated with FTR. To limit this inconvenience, we excluded those trajectories (i.e. combinations of procedures and postoperative complications) with fewer than 10 procedures during the period. As a result of the limited numbers within each pathway, it was difficult to account for the possibility that death often occurs after a cascade of several complications. This underlines that identifying trajectories to FTR is actually harder than one might expect. In the study by Wakeam *et al.*, they demonstrated that the risk of a complication was highest among patients who had already developed a previous complication [23], showing that the FTR pathway is complex and associated with numerous interconnecting factors. In addition, according to the definition of FTR, it is not possible to discriminate between deaths caused by the complication and deaths due to other causes. Although we only included one postoperative complication in our trajectories, we believe that this gave sufficient information on the progression from complications to death, and investigating the impact on several complications, including reoperations, will be interesting for future studies. In addition, there is always a tradeoff between too ‘general’ (high precision and less specific) and too ‘complex’ (less precise and more informative) trajectory pattern.

Third, there was a risk of including complications that were not related to the index surgical procedure. Toward this, conditions counted as complications may have been present before surgery, as the NPR does not have a code for the condition being present on admission. We acknowledge that this might be the case for ERCP and open colon resections, as sepsis might be present at the time of the procedure and not occur after the procedure. In a recent study in two Norwegian hospitals, the presence of surgical complications in inpatient administrative systems was found to have a sensitivity and specificity of 56% and 95%, respectively, and 76% and 65% after exclusion of conditions present on admission [24].

## Conclusions

This study has shown that distinct surgical procedures exist with subsequent complications that are highly associated with FTR. Sepsis and respiratory failure are the two most common complications in high-volume trajectories. These trajectories should be targeted in national quality improvement programs to decrease FTR at a national level.

## Supplementary Material

mzac084_Supp

## Data sharing statement

Individual-level data of patients included in this manuscript after de-identification are considered sensitive and will not be shared. The study method and statistical analyses are all described in detail in the Methods section and throughout the manuscript.
