# Supplementary material for: Abdominal surgical trajectories associated with failure to rescue. A nationwide analysis
Source: Int J Qual Health Care. 2022 Oct 26;34(4):mzac084. doi: 10.1093/intqhc/mzac084 (PMC9670749; doi:10.1093/intqhc/mzac084)
Supplement: mzac084_Supp [file mzac084_supp.zip › suppl_data/Revision_Online Supplement tables.docx]

Supplementary tables for the paper

Abdominal Surgical Trajectories Associated with Failure to Rescue. A Nationwide Analyses

By Skyrud et al., 2022

**Table of contents (brief titles):**

| Figure S1. Flowchart of the study population |  |
| --- | --- |
|  |  |
| Table S1. 30-day FTR for all postoperative complications by surgical specialty |  |
| Table S2. 30-day FTR for all surgical procedure type by surgical specialty |  |
|  |  |
| Table S3. Patient characteristics for the two pathways with highest FTR-V within each the surgical specialty urological and gynaecological surgery. |  |
|  |  |
| Table S4. The five highest FTR-R surgical pathways by surgical specialty |  |
|  |  |
| Table S5. ICD-10 diagnosis codes for complications after abdominal surgery |  |
| Table S6. NCSP codes for abdominal surgery |  |
|  |  |
|  |  |

Figure S1. Flowchart of the study population


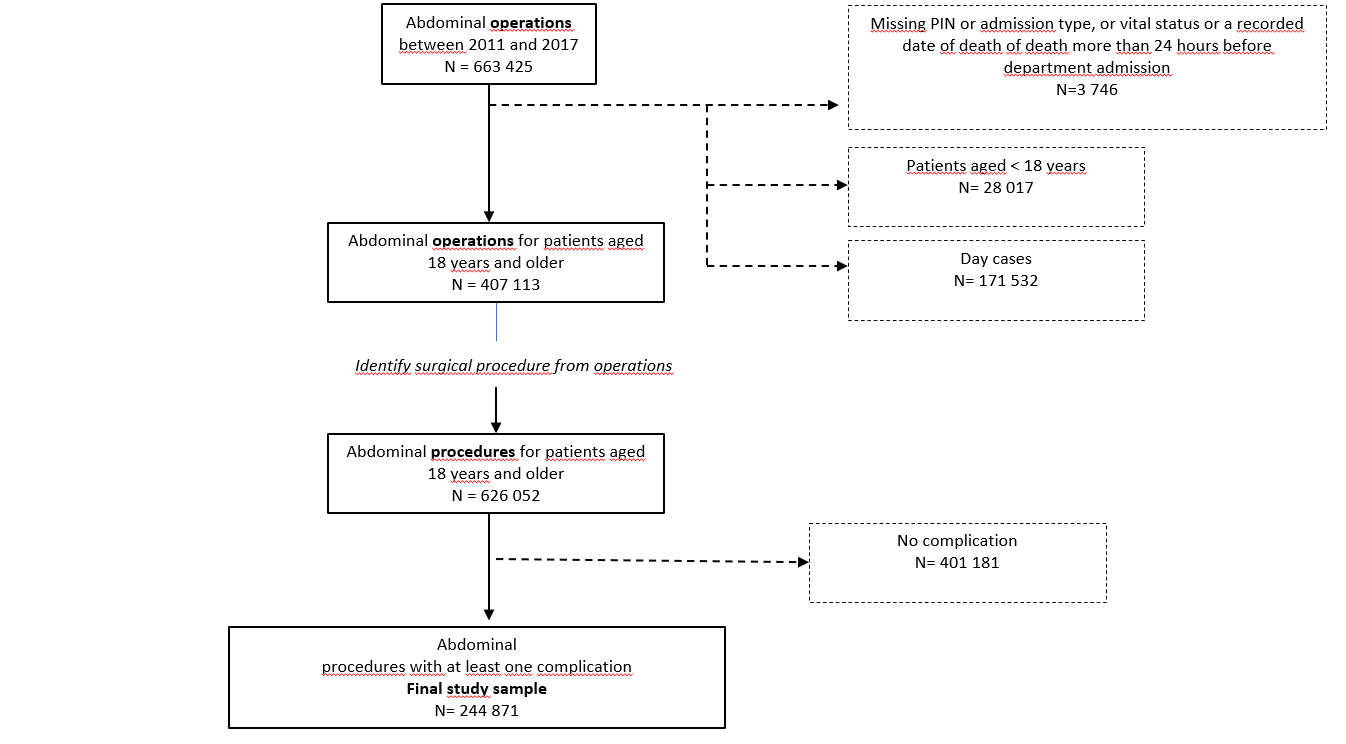


Table S1. Total Number of Procedures with Postoperative Complications, the Number of Deaths Within 30 Days, and the 30-Day FTR, in Total and Within Each Specialty

| Postoperative Complication | Total Number of Procedures | 30-Day FTR (Number of Deaths) | | | | |
| --- | --- | --- | --- | --- | --- | --- |
|  | **All** | **All**^d^ | **Gastrointestinal** | **Vascular** | **Gynecology** | **Urology** |
| Any complication | 224 871 | 7.6 (17,090) | 10.0 (22,487) | 9.8 (22,037) | 1.0 (2,249) | 2.5 (5,622) |
| Cardiac arrest | 1 202 | 45.8 (551) | 47.0 (358) | 41.8 (74) | 14.8 (4) | 47.1 (40) |
| Acute ischemia of the bowel | 2 342 | 25.7 (603) | 24.9 (467) | 34.9 (73) | 25.0 (4) | 21.4 (6) |
| Respiratory failure | 7 970 | 23.5 (1871) | 24.9 (1455) | 23.8 (159) | 8.8 (14) | 17.0 (108) |
| Sepsis | 14 331 | 17.8 (2554) | 21.6 (2029) | 25.7 (118) | 5.5 (14) | 7.6 (233) |
| Stroke and intracranial bleeding | 3 008 | 16.8 (504) | 17.9 (345) | 19.3 (75) | 4.4 (4) | 12.6 (53) |
| Acute myocardial infarction | 896 | 14.4 (129) | 19.6 (94) | 11.0 (21) | 3.4 (1) | 6.7 (10) |
| Acute kidney failure | 5 666 | 13.7 (778) | 17.7 (450) | 26.2 (116) | 11.1 (10) | 6.1 (134) |
| Congestive heart failure | 8 693 | 12.0 (1047) | 15.2 (672) | 15.5 (243) | 3.5 (12) | 4.3 (83) |
| Delirium and somnolence | 510 | 7.8 (40) | 10.7 (33) | 6.2 (2) | 3.0 (1) | 3.8 (4) |
| Other infections^a^ | 819 | 6.3 (52) | 7.3 (37) | 6.2 (5) | 5.9 (2) | 3.5 (4) |
| Embolism and arterial thrombosis | 5 268 | 6.0 (314) | 7.4 (114) | 7.4 (169) | 1.5 (3) | 4.2 (19) |
| Other surgical complications^b^ | 26 503 | 5.3 (1403) | 6.7 (959) | 15.6 (182) | 0.4 (7) | 2.2 (110) |
| Malnutrition | 8 659 | 5.0 (432) | 5.6 (361) | 4.3 (17) | 3.4 (4) | 5.2 (45) |
| Surgical wound rupture | 4 835 | 4.4 (213) | 5.8 (189) | 2.0 (4) | 0.0 (0) | 3.6 (13) |
| Disorders in the fluid/electrolytes balance | 6 967 | 4.3 (303) | 4.9 (247) | 5.1 (16) | 4.7 (8) | 3.0 (21) |
| Minor respiratory complications^c^ | 9 033 | 3.3 (299) | 5.1 (228) | 3.5 (15) | 0.4 (5) | 1.5 (27) |
| Arrhythmia | 22 645 | 3.1 (697) | 4.6 (472) | 4.8 (112) | 0.5 (7) | 1.0 (77) |
| Postoperative bowel obstruction | 1 700 | 2.3 (39) | 2.5 (33) | 0.0 (0) | 0.0 (0) | 1.9 (2) |
| Angina pectoris | 3 710 | 2.1 (77) | 3.6 (50) | 2.3 (14) | 0.0 (0) | 0.6 (8) |
| Surgical site and infusion or implant related | 11 876 | 1.7 (204) | 2.4 (167) | 2.1 (12) | 0.2 (1) | 0.6 (13) |
| Surgical bleeding | 8 527 | 1.5 (129) | 2.4 (60) | 2.4 (36) | 0.0 (0) | 0.8 (14) |
| Anesthesia complications | 1 647 | 1.3 (22) | 1.7 (13) | 6.0 (5) | 0.0 (0) | 0.3 (1) |
| Mechanical and other implant complication | 1 408 | 1.3 (19) | 3.2 (10) | 0.0 (0) | 0.0 (0) | 0.6 (5) |
| Urinary tract infection | 18 066 | 1.0 (183) | 2.3 (82) | 0.3 (2) | 0.5 (8) | 0.8 (82) |
| Fall | 30 | 0.0 (0) | 0.0 (0) | 0.0 (0) | 0.0 (0) | 0.0 (0) |

The 30-day FTR rate for each postoperative complication was calculated by the number of deaths divided by the total number of procedures with the given postoperative complication. ICD-10 codes for each complication can be found in supplement table 4.

^a^Other infections include meningitis, peri- and endocarditis, gastroenteritis

^b^Other surgical complications include circulatory collapse, unintended punctures or lacerations, unspecified kidney failure, and complications of surgery not classified

^c^Minor respiratory complications include asthma, pleura effusion and dyspnea

^d^Includes also procedures with more than one specialty, therefore adding number of deaths from each specialty may be smaller

Table S2. Total Number of Procedures with Type of Surgery, the Number of Deaths Within 30 Days, and the 30-Day FTR by surgical specialty.

| Surgical specialty | Type of surgery | No of procedures | No of deaths within 30 days | 30-day FTR rate (%) |
| --- | --- | --- | --- | --- |
| Vascular | Embolectomy visceral arteries | 165 | 58 | 35.2 |
|  | Suprarenal bypass | 213 | 34 | 16.0 |
|  | Infrarenal abdominal aorta surgery (infrarenal AAA) | 2 645 | 358 | 13.5 |
|  | Suprarenal/visceral surgery | 118 | 14 | 11.9 |
|  | Visceral reimplantation | 265 | 27 | 10.2 |
|  | Aneeurysm in suprarenal arteries | 76 | 7 | 9.2 |
|  | Femoral artery surgery | 8 892 | 786 | 8.8 |
|  | Bypass aortoiliakal | 708 | 57 | 8.1 |
|  | Aorta plasty | 132 | 6 | 4.5 |
| Gastrointestinal | Colon stenting | 197 | 39 | 19.8 |
|  | Total colectomy | 1 332 | 215 | 16.1 |
|  | Minor surgery stomach* | 5 666 | 824 | 14.5 |
|  | Major surgery stomach** | 1 865 | 270 | 14.5 |
|  | Stoma formation | 6 485 | 876 | 13.5 |
|  | Open procedures small intestine | 7 158 | 931 | 13.0 |
|  | Open colon resections | 9 535 | 1 080 | 11.3 |
|  | Anastomosis formation | 1 532 | 154 | 10.1 |
|  | Cholecystectomy | 1 467 | 131 | 8.9 |
|  | Endoscopic Retrograde Cholangiography (ERCP) | 11 004 | 982 | 8.9 |
|  | Abdominal wall surgery | 1 153 | 98 | 8.5 |
|  | Freeing of adhesions/omentectomy | 6 999 | 537 | 7.7 |
|  | Splenectomy | 912 | 68 | 7.5 |
|  | Pancreas surgery | 1 463 | 90 | 6.2 |
|  | Laparoscopy | 6 867 | 403 | 5.9 |
|  | Inguinal and femoral hernia repair (open) | 2 280 | 110 | 4.8 |
|  | Laparoscopic procedures small intestine | 188 | 9 | 4.8 |
|  | Closure or stoma revision | 2 467 | 104 | 4.2 |
|  | Appendectomy | 1 757 | 69 | 3.9 |
|  | Other transanal surgery | 997 | 39 | 3.9 |
|  | Open rectal procedures | 3 431 | 133 | 3.9 |
|  | Incisional hernia and abdominal wall reconstruction | 2 215 | 81 | 3.7 |
|  | Ant reflux operation/ paraoesophageal hernia | 522 | 17 | 3.3 |
|  | Laparoscopic colon resections | 3 409 | 99 | 2.9 |
|  | Liver surgery | 1 391 | 40 | 2.9 |
|  | esophagectomy | 259 | 5 | 1.9 |
|  | Laparoscopic rectal procedures | 1 050 | 20 | 1.9 |
|  | Laparoscopic appendectomy | 2 036 | 21 | 1.0 |
|  | Rectopexy | 188 | 1 | 0.5 |
|  | Laparoscopic hernia repair | 849 | 4 | 0.5 |
| Urology | External drainage of pyelum | 560 | 81 | 14.5 |
|  | Exploration of the kidney | 24 | 3 | 12.5 |
|  | Other ureter surgery*** | 141 | 9 | 6.4 |
|  | Other surgery | 18 | 1 | 5.6 |
|  | Retroperitoneal surgery | 349 | 19 | 5.4 |
|  | Excision kidney | 1 636 | 60 | 3.7 |
|  | Ureter stents | 12 292 | 431 | 3.5 |
|  | Biopsy kidney | 242 | 8 | 3.3 |
|  | Vesical surgery inclusive reconstruction | 10 479 | 297 | 2.8 |
|  | Percutaneous drainage of ureter | 1 479 | 40 | 2.7 |
|  | Reconstruction and anastomosis ureter | 1 001 | 25 | 2.5 |
|  | Excision/resection of the ureter | 126 | 3 | 2.4 |
|  | Foreign body removal ureter | 42 | 1 | 2.4 |
|  | Urethral surgery | 102 | 2 | 2.0 |
|  | Reconstruction urethra | 372 | 6 | 1.6 |
|  | Reconstruction’s kidney/pyelum | 135 | 2 | 1.5 |
|  | Resection kidney | 823 | 9 | 1.1 |
|  | Prostate surgery | 7 883 | 66 | 0.8 |
|  | Ureter stone removal | 1 868 | 12 | 0.6 |
|  | Surgery for incontinence | 205 | 1 | 0.5 |
|  | Removal of stones form the kidney/ureter | 1 932 | 7 | 0.4 |
|  | Exploration of the ureter | 55 | 0 | 0.0 |
|  | Removal of foreign body kidney/ureter | 25 | 0 | 0.0 |
|  | Sphincter prosthesis | 742 | 0 | 0.0 |
| Gynaecology | Reconstruction uterus | 74 | 2 | 2.7 |
|  | Ovarian surgery | 4 414 | 106 | 2.4 |
|  | Uterus surgery | 5 897 | 98 | 1.7 |
|  | Cervix surgery | 384 | 2 | 0.5 |
|  | Tube surgery | 714 | 2 | 0.3 |
|  | Vagina | 2 652 | 3 | 0.1 |
|  | Incontinence surgery | 675 | 0 | 0.0 |
|  | Sterilization | 99 | 0 | 0.0 |

*Minor surgery stomach; including gastrotomy, percutaneous gastrostomy.

**Major surgery of the stomach; including gastrectomy, gastrojejunostomy, gastroplasty, duodenotomy.

***Other ureter surgery; including other percutaneous/ transluminal endoscopic operation on kidney or pelvis of kidney. Other ureter surgery; including other percutaneous/ transluminal endoscopic operation on ureter

S-Table 3. Patient characteristics for the two pathways with highest FTR-V within each the surgical specialty urological and gynaecological surgery.

| **Type of surgery and postoperative complication** | **Ureter stents and sepsis**  **(Urological surgery)** | | | **Ureter stents and acute kidney failure**  **(Urological surgery)** | | | **Ovarian surgery and respiratory failure**  **(Gynaecological surgery)** | | | **Uterus surgery and respiratory failure**  **(Gynaecological surgery)** | | |
| --- | --- | --- | --- | --- | --- | --- | --- | --- | --- | --- | --- | --- |
|  | **No deaths within 30-days** | **Deaths within 30 days** | **P-value*** | **No deaths within 30-days** | **Deaths within 30 days** | **P-value*** | **No deaths within 30-days** | **Deaths within 30 days** | **P-value*** | **No deaths within 30-days** | **Deaths within 30 days** | **P-value*** |
| Number of procedures | 1 462 | 108 |  | 1 094 | 64 |  | 81 | 17 |  | 102 | 15 |  |
| Age, mean | 66.2 | 74.5 | 0.05 | 68.1 | 75.6 | <0.001 | 65.6 | 75.7 | n.s | 64.3 | 75.1 | n.s |
| Elective admissions. % | 24.6 | 25.0 | n.s | 24.5 | 14.1 | n.s | 60.5 | 64.7 | n.s | 59.8 | 66.7 | n.s |
| Male, % | 45.6 | 41.7 | n.s | 63.7 | 64.1 | n.s | 0.0 | 0.0 |  | 0.0 | 0.0 |  |
| Charlson index. mean | 1.9 | 3.6 | <0.001 | 2.1 | 3.9 | n.s | 1.1 | 2.2 | n.s | 1.1 | 2.5 | n.s |
| **Hospital size** |  |  |  |  |  |  |  |  |  |  |  |  |
| Large | 776 | 70 | n.s | 671 | 36 | n.s | 26 | 4 | n.s | 43 | 3 | n.s |
| Medium | 437 | 21 |  | 294 | 18 |  | 51 | 12 |  | 49 | 11 |  |
| Small | 160 | 11 |  | 60 | 5 |  | - | - |  | 10 | 2 |  |
| **Most common comorbidities (%)** |  |  |  |  |  |  |  |  |  |  |  |  |
| None | 36.0 | 17.8 |  | 33.4 | 10.7 |  | 59.6 | 31.8 |  | 53.5 | 30.0 |  |
| Any malignancy^a^ | 23.6 | 31.6 |  | 24.8 | 36.6 |  | 23.6 | 31.8 |  | 19.8 | 35.0 |  |
| Chronic pulmonary disease | 7.1 | 4.0 |  | 4.4 | 5.4 |  | 5.6 | 13.6 |  | 13.8 | 10.0 |  |
| Renal disease | 13.1 | 13.2 |  | 17.0 | 18.8 |  | 2.3 |  |  | 1.7 |  |  |
| Congestive heart failure | 4.1 | 6.9 |  | 4.9 | 5.4 |  | 1.1 |  |  | 3.4 | 5.0 |  |
| Metastatic disease | 8.7 | 19.0 |  | 10.0 | 17.0 |  | 5.6 | 13.6 |  | 4.3 | 15.0 |  |
| * P-value after fitting a logistic regression. adjusted for multiple testing | | | | | | | | | | | | |

Table S4. The five combinations of procedure type and postoperative complications associated with highest risk of failure to rescue (FTR-R).

| Surgical specialty | Surgical procedure | Postoperative complication | No of procedures | No of deaths | 30-day FTR |
| --- | --- | --- | --- | --- | --- |
| Vascular | *Highest risk* | |  |  |  |
|  | Embolectomy visceral arteries | Sepsis | 27 | 22 | 81.5 |
|  | Embolectomy visceral arteries | Acute kidney failure | 17 | 10 | 58.8 |
|  | Infrarenal abdominal aorta surgery | Cardiac arrest | 42 | 19 | 45.2 |
|  | Visceral reimplantation | Acute ischemia of the bowel | 23 | 10 | 43.5 |
|  | Embolectomy visceral arteries | Respiratory failure | 17 | 7 | 41.2 |
| Gastrointestinal | *Highest risk* | |  |  |  |
|  | Liver surgery | Cardiac arrest | 16 | 12 | 75.0 |
|  | Pancreas surgery | Acute ischemia of the bowel | 11 | 7 | 63.6 |
|  | Major surgery stomach | Cardiac arrest | 36 | 22 | 61.1 |
|  | Open procedures small intestine | Cardiac arrest | 156 | 76 | 48.7 |
|  | Open colon resections | Cardiac arrest | 169 | 82 | 48.5 |
| Urology | *Highest risk* | |  |  |  |
|  | Vesical surgery | Cardiac arrest | 32 | 21 | 65.6 |
|  | Ureter stents | Cardiac arrest | 31 | 16 | 51.6 |
|  | Prostata surgery | Cardiac arrest | 16 | 8 | 50.0 |
|  | Excision kidney | Cardiac arrest | 16 | 7 | 43.8 |
|  | Extrenal drainage of pyelum | Malnutrition | 18 | 7 | 38.9 |
| Gynecology | *Highest risk* | |  |  |  |
|  | Ovarial surgery | Acute ischemia of the bowel | 18 | 7 | 38.9 |
|  | Uterus | Acute ischemia of the bowel | 13 | 4 | 30.8 |
|  | Ovarial surgery | Cardiac arrest | 14 | 3 | 21.4 |
|  | Ovarial surgery | Respiratory failure | 137 | 23 | 16.8 |
|  | Uterus | Cardiac arrest | 19 | 3 | 15.8 |

Table S5. ICD-10 diagnosis codes for complications after abdominal surgery

| Complications | ICD-10 |
| --- | --- |
| *Respiratory complications* |  |
| Pneumonia | J12-J18 |
| Respiratory failure | J96, R09.2 |
| Minor respiratory complications  asthma, pleura-effusion, dyspnoea | J45, J46, J80, J82-84, J90, J91, J93, J95, R06, R09.0-1, R09.3-8 |
| *Cardiac complications* |  |
| Cardiac arrest | I46, R96 |
| Arrhythmia | I44, I45, I47, I48, I49 |
| Congestive heart failure | I50, I51, J81 |
| Acute myocardial infarction | I21-I24 |
| Angina pectoris | I20 |
| *Infections* |  |
| Sepsis | A02.1, A20.7, A40, A41, A42.7, B37.7, R57.2, R65.0, R65.1 |
| Surgical site and infusion or implant related | T80.2, T81.4, T82.7, T83.5-6, T85.7 |
| Urinary tract | N30, N39.0 |
| Other infections  meningitis, peri- and endocarditis, gastroenteritis | A04, G00, I30, I31, I38-I40, Y95 |
| *Other surgical complications* |  |
| Surgical wound rupture | T81.3 |
| Nervous system complication  Delirium and somnolence | F05, I640, R29, R40, R41 |
| Stroke and intracranial bleeding | I61-I64 |
| Surgical bleeding | T80.3-4, T81.0, T82.8 |
| Embolism and arterial thrombosis,  Arterial-, venous-, lung- and air embolism | I26, I74, I80, T80.0, T81.7 |
| Mechanical and other implant complication | T82.3, T82.5, T83.0-2, T83.4, T83.8-9,  T85.5-6, T85.8-9 |
| Anaesthesia complications | T4n, T41, T50,  T88.2-9, Y48 |
| Acute kidney failure | N17 |
| Other complications  Circulatory collapse, unintended punctures  or lacerations, unspecified kidney failure,  complications following surgical and  medical procedures and complications to surgery not classified | K25.0-2, K26.0-2, L89.0-9, N19,  R57, R57.0-1, R57.8-9, T78.2, T81.1-2, T81.5-6, T81.8-9 |
| Acute ischemia of the bowel | K55.0, K55.8, K55.9 |
| Postoperative bowel obstruction | K91.3 |
| Fall | W0n |
| Malnutrition | E40-E46, E50-E64 |
| Disorders in the fluid/electrolytes balance | E87 |

Table S6. NCSP codes for abdominal surgery

| NCSP code | Text |
| --- | --- |
|  | **Abdominal wall surgery** |
| JAA00 | Incision of abdominal wall |
| JAA10 | Excision of lesion of abdominal wall |
| JAA11 | Laparoscopic biopsy or excision of lesion of abdominal wall |
| JAA96 | Other local operation on abdominal wall |
|  | Inguinal and femoral hernia repair (open) |
| JAB00 | Division and ligature of inguinal hernia sac |
| JAB10 | Repair of inguinal hernia |
| JAB20 | Repair of inguinal hernia using using graft |
| JAB30 | Repair of inguinal hernia using prosthetic material |
| JAC10 | Repair of femoral hernia |
| JAC30 | Repair of femoral hernia using prosthetic material |
|  | **Laparoscopic hernia repair** |
| JAB11 | Laparoscopic repair of inguinal hernia |
| JAC11 | Laparoscopic repair of femoral hernia |
|  | Incisional hernia and abdominal wall reconstruction |
| JAD10 | Repair of incisional hernia |
| JAD30 | Repair of incisional hernia using prosthetic material |
| JAE10 | Repair of epigastric hernia |
| JAG00 | Repair of other hernia |
| JAG01 | Laparoscopic repair of other hernia |
| JAG30 | Reconstruction of abdominal wall using flap |
| JAG60 | Reconstruction of abdominal wall using prosthetic material |
|  | **Laparotomy** |
| *JAH00* | *Laparotomy* |
| *JAH20* | *Staging laparotomy* |
| *JAK00* | *Laparotomy and drainage of peritoneal cavity* |
| *JAK03* | *Laparotomy and peritoneal irrigation* |
| JAK10 | Laparotomy and insertion of peritoneal dialysis catheter |
| JAL10 | Laparotomy and removal of foreign body |
| JFG50 | Laparotomy with revision of enterostomy or colostomy |
|  | **Laparoscopy** |
| *JAH01* | *Laparoscopy* |
| *JAH21* | *Staging laparoscopy* |
| *JAK01* | *Laparoscopic drainage of peritoneal cavity* |
| *JAK04* | *Laparoscopy and peritoneal irrigation* |
| JAP01 | Laparoscopic freeing of adhesions in the peritoneal cavity |
| JFK97 | Other laparoscopic operation on adhesions in intestinal obstruction |
|  | **Freeing of adhesions/omentectomy** |
| JAL00 | Biopsy of peritoneum |
| JAL20 | Excision or destruction of lesion of peritoneum |
| JAL30 | Omentectomy |
| JAM00 | Transposition of omentum |
| JAP00 | Freeing of adhesions in the peritoneal cavity |
| JFK00 | Division of adhesive band in intestinal obstruction |
| JFK10 | Freeing of adhesions in intestinal obstruction |
|  | **Antireflux operation/ paraoesophageal hernia** |
| JBB00 | Repair of paraoesophageal hernia |
| JBB01 | Laparoscopic repair of paraoesophageal hernia |
| JBC00 | Gastro-oesophageal antireflux operation |
| JBC01 | Laparoscopic gastro-oesophageal antireflux operation |
| JBW96 | Other transabdominal operation on diaphragm or operation for gastro-oesophageal reflux |
|  | **Oesophagectomy** |
| JCC00 | Transhiatal partial oesophagectomy without interposition |
| JCC10 | Transthoracic partial oesophagectomy without interposition |
| JCC30 | Transthoracic partial oesophagectomy with interposition of intestine |
|  | **Minor surgery stomach** |
| JDA00 | Gastrotomy |
| JDA60 | Closure of perforated ulcer of stomach |
| JDA61 | Laparoscopic closure of perforated ulcer of stomach |
| JDA63 | Local excision of lesion of stomach |
| JDB00 | Gastrostomy |
| JDB10 | Percutaneous gastrostomy |
|  | **Major surgery of stomach** |
| JDC10 | Partial gastrectomy and gastrojejunostomy |
| JDC11 | Laparoscopic partial gastrectomy and gastrojejunostomy |
| JDC20 | Partial gastrectomy and Roux-en-Y reconstruction |
| JDC40 | Partial gastrectomy and oesophagogastrostomy |
| JDD00 | Total gastrectomy and Roux-en-Y oesophagojejunostomy |
| JDE00 | Gastrojejunostomy |
| JDE10 | Conversion of gastrojejunostomy to Roux-en-Y anastomosis |
| JDF00 | Gastroplasty |
| JDF10 | Gastric bypass |
| JDG00 | Truncal vagotomy |
| JDG97 | Other laparoscopic vagotomy |
| JDH00 | Duodenotomy |
| JDH61 | Laparoscopic pyloromyotomy |
| JDH63 | Pyloroplasty |
| JDH70 | Closure of perforated ulcer of duodenum |
|  | **Appendectomy** |
| JEA00 | Appendectomy |
| JEA10 | Appendectomy with drainage |
|  | **Laparoscopic appendectomy** |
| JEA01 | Laparoscopic appendectomy |
|  | **Colon stenting** |
| JFA68 | Endoscopic insertion of prosthetic tube into colon |
|  | **Open procedures on small intestine** |
| JFA60 | Stricturoplasty in small intestine |
| JFA70 | Suture of small intestine |
| JFA73 | Excision of lesion of small intestine |
| JFA76 | Closure of fistula of small intestine |
| JFA83 | Excision of lesion of colon |
| JFB00 | Partial resection of small intestine |
| JFB10 | Reversal of segment of small intestine |
| JFB96 | Other partial excision of intestine |
| JFL00 | Open reduction of intussusception of intestine |
| JFW96 | Other operation on intestine |
|  | **Laparascopic procedures on small intestine** |
| JFA97 | Other laparoscopic local operation on intestine |
| JFB01 | Laparoscopic partial resection of small intestine |
| JFB97 | Other laparoscopic partial excision of intestine |
|  | **Open colon resections** |
| JFB20 | Ileocaecal resection |
| JFB30 | Right hemicolectomy |
| JFB33 | Other resection comprising small intestine and colon |
| JFB40 | Resection of transverse colon |
| JFB43 | Left hemicolectomy |
| JFB46 | Resection of sigmoid colon |
| JFB50 | Other resection of colon |
| JFB60 | Resection of sigmoid colon with end colostomy |
|  | **Laparascopic colon resections** |
| JFB21 | Laparoscopic ileocaecal resection |
| JFB31 | Laparoscopic right hemicolectomy |
| JFB44 | Laparoscopic left hemicolectomy |
| JFB47 | Laparoscopic resection of sigmoid colon |
| JFB51 | Other laparoscopic resection of colon |
| JFB54 | Laparoscopiic resection of sigmoid colon sigmoideum with partial resection of rectum |
| JFB61 | Laparoscopic resection of sigmoid colon with end colostomy and closure of distal stump |
| JFH11 | Laparoscopic total colectomy and ileostomy |
|  | **Anastomosis formation** |
| JFC00 | Entero-enterostomy |
| JFC10 | Ileotransversostomy |
| JFC20 | Other enterocolostomy |
| JFC30 | Colo-colostomy |
|  | **Stoma formation** |
| JFF00 | Catheter enterostomy |
| JFF10 | Loop enterostomy |
| JFF11 | Laparoscopic loop enterostomy |
| JFF13 | Terminal enterostomy |
| JFF20 | Caecostomy |
| JFF23 | Transversostomy |
| JFF26 | Sigmoidostomy |
| JFF27 | Laparoscopic sigmoidostomy |
|  | **Closure or stoma revision** |
| JFF96 | Other exteriorisation of intestine or creation of intestinal stoma |
| JFG00 | Closure of loop enterostomy without resection |
| JFG10 | Closure of loop colostomy without resection |
| JFG20 | Closure of enterostomy with resection of exteriorised loop |
| JFG23 | Closure of terminal enterostomy with anastomosis to small intestine |
| JFG26 | Closure of terminal enterostomy with anastomosis to colon |
| JFG30 | Closure of colostomy with resection of exteriorised loop |
| JFG36 | Closure of terminal colostomy with anastomosis to rectum |
| JFG40 | Revision of enterostomy or colostomy without laparotomy |
| JFG96 | Other operation on intestinal stoma or pouch |
|  | **Total colectomy** |
| JFG73 | Excision of ileal pelvic pouch |
| JFH00 | Total colectomy and ileorectal anastomosis |
| JFH10 | Total colectomy and ileostomy |
| JFH20 | Proctocolectomy and ileostomy |
| JFH30 | Total colectomy, mucosal proctectomy and ileoanal anastomosis without ileostomy |
| JFH33 | Total colectomy, mucosal proctectomy, ileoanal anastomosis and ileostomy |
| JFH40 | Proctocolectomy and continent ileostomy |
| JFH96 | Other total colectomy |
|  | **Open rectal procedures** |
| JGA05 | Endoscopic polypectomy in rectum |
| JGA60 | Suture of rectum |
| JGA73 | Transanal excision of lesion of rectum |
| JGA75 | Endoscopic microsurgical excision of lesion of rectum |
| JGA96 | Other proctotomy or local operation on rectum |
| JGA98 | Other transluminal endoscopic local operation on rectum |
| JGB00 | Partial proctectomy and colorectal or coloanal anastomosis |
| JGB10 | Partial proctectomy and end colostomy |
| JGB30 | Abdominoperineal excision of rectum |
| JGB50 | Mucosal proctectomy and ileoanal anastomosis |
| JGB96 | Other proctectomy or excision of rectum |
|  | **Laparascopic rectal procedures** |
| JGB01 | Laparoscopic partial proctectomy and colorectal or coloanal anastomosis |
| JGB04 | Laparoscopic partial proctectomy with partial excision of mesorectum |
| JGB07 | Laprascopic low anterior resection of rectum |
| JGB31 | Laparoscopic and perineal excision of rectum |
| JGB11 | Laparoscopic partial proctectomy and end colostomy |
| JGB97 | Other laparoscopic proctectomy or excision of rectum |
|  | **Rektopexy** |
| JGC00 | Rectopexy |
| JGC01 | Laparoscopic rectopexy |
|  | **Other transanal surgery** |
| JGC20 | Transanal suture |
| JGC96 | Other reconstructive operation on rectum |
| JGW98 | Other transluminal endoscopic operation on rectum |
| JHA00 | Anal or perianal incision |
| JHC00 | Suture of anal sphincter |
| JHC10 | Reconstruction of anal sphincter without graft |
|  | **Liver surgery** |
| JJA00 | Exploration of liver |
| JJA31 | Laparoscopic fenestration of cyst of liver |
| JJA43 | Destruction of lesion of liver |
| JJB00 | Wedge resection of liver |
| JJB10 | Atypical resection of liver |
| JJB20 | Excision of single segment of liver |
| JJB30 | Excision of two segments of liver |
| JJB40 | Excision of segments II, III and IV of liver |
| JJB50 | Excision of segments V, VI, VII and VIII of liver |
| JJB53 | Excision of segments IV,V, VI, VII and VIII of liver |
|  | **Cholecystectomy** |
| JKA00 | Cholecystotomy |
| JKA10 | Cholecystostomy |
| JKA13 | Percutaneous cholecystostomy |
| JKA20 | Cholecystectomy |
| JKA11 | Laparoscopic cholecystostomy |
|  | **Endoscopic retrograde cholangiography (ERCP)** |
| JKB30 | Percutaneous transhepatic biliary drainage |
| JKE00 | Transduodenal papillotomy |
| JKE02 | Transduodenal endoscopic incision of common bile duct orifice |
| JKE12 | Endoscopic extraction of calculus from bile duct |
| JKE18 | Endoscopic insertion of stent into bile duct |
| JKT10 | Extracorporeal shock wave lithotripsy of biliary duct |
|  | **Pancreas surgery** |
| JLB00 | Incision of pancreas |
| JLB96 | Other incision, drainage or dilatation of pancreas |
| JKD10 | Anastomosis of bile duct to duodenum |
| JKD20 | Anastomosis of bile duct to jejunum |
| JLC00 | Excision of lesion of pancreas |
| JLC10 | Distal pancreatectomy |
| JLC20 | Total pancreatectomy |
| JLC30 | Pancreatoduodenectomy |
| JLC40 | Total pancreatoduodenectomy |
| JLC50 | Atypical pancreatectomy |
| JLC96 | Other pancreatectomy |
| JLD10 | Anastomosis of pancreatic pseudocyst to stomach |
| JLW98 | Other transluminal endoscopic operation on pancreas |
|  | **Splenectomy** |
| JMA10 | Transabdominal total splenectomy |
| JMA11 | Laparoscopic total splenectomy |
|  | **Exploration of the kidney** |
| KAA00 | Exploration of kidney |
| KAA01 | Percutaneous endoscopic exploration of kidney |
| KAA20 | Exploratory nephrotomy |
| KAA21 | Percutaneous endoscopic exploratory nephrotomy |
| KAA30 | Exploratory pyelotomy |
| KAA31 | Percutaneous nephroscopy |
| KAA96 | Other exploration of kidney or pelvis of kidney |
| KAA97 | Other percutaneous endoscopy of kidney or pelvis of kidney |
|  | **Biopsy kidney** |
| KAB00 | Biopsy of kidney or pelvis of kidney |
| KAB01 | Percutaneous endoscopic biopsy of kidney or pelvis of kidney |
|  | **Excision of kidney** |
| KAC00 | Nephrectomy |
| KAC01 | Percutaneous endoscopic nephrectomy |
| KAC20 | Nephroureterectomy |
| KAC21 | Percutaneous endoscopic nephroureterectomy |
|  | **Resection of kidney** |
| KAD00 | Partial nephrectomy |
| KAD01 | Percutaneous endoscopic partial nephrectomy |
| KAD11 | Percutaneous endoscopic heminephrectomy |
| KAD40 | Partial excision of pelvis of kidney |
| KAD41 | Percutaneous endoscopic partial excision of pelvis of kidney |
| KAD50 | Destruction of tumour of pelvis of kidney |
| KAD51 | Percutaneous endoscopic destruction of tumour of pelvis of kidney |
| KAD52 | Retrograde ureteronephroscopic destruction of tumour of pelvis of kidney |
| KAD56 | Destruction of lesion of renal parenchyma |
| KAD60 | Percutaneous destruction of lesion of renal parenchyma |
| KAD96 | Other partial excision of kidney or pelvis of kidney |
| KAD97 | Other percutaneous endoscopic partial excision or destruction of tumour of kidney or pelvis of kidney |
| KAD98 | Other transluminal endoscopic partial excision of pelvis of kidney |
|  | **Removal of stones form the kidney/ureter** |
| KAE00 | Nephrolithotomy |
| KAE01 | Nephroscopic nephrolithotomy |
| KAE10 | Pyelolithotomy |
| KAE11 | Nephroscopic pyelolithotomy |
| KAE12 | Retrograde ureteronephroscopic lithotripsy in pelvis of kidney |
| KAE96 | Other removal of calculus from kidney or pelvis of kidney |
| KAE97 | Other percutaneous endosopic removal of calculus from kidney or pelvis of kidney |
| KAE98 | Other transluminal endosopic removal of calculus from kidney or pelvis of kidney |
|  | **Removal of foreign body from kidney/ureter** |
| KAF00 | Removal of foreign body from kidney |
| KAF01 | Percutaneous endoscopic removal of foreign body from kidney |
| KAF10 | Removal of foreign body from pelvis of kidney |
| KAF11 | Percutaneous endoscopic removal of foreign body from pelvis of kidney |
| KAF12 | Transluminal removal of foreign body from pelvis of kidney |
|  | **Reconstruction of kidney/pyelum** |
| KAH00 | Suture of kidney |
| KAH01 | Percutaneous endoscopic suture of kidney |
| KAH10 | Suture of pelvis of kidney |
| KAH11 | Percutaneous endoscopic suture of pelvis of kidney |
| KAH30 | Pyeloureteroplasty without division of ureteropelvic junction |
| KAH31 | Percutaneous endoscopic pyeloureteroplasty without division of ureteropelvic junction |
| KAH40 | Pyeloureteroplasty with division of ureteropelvic junction |
| KAH41 | Percutaneous endoscopic pyeloureteroplasty with division of ureteropelvic junction |
| KAH50 | Ureterocalyceal anastomosis |
| KAH51 | Percutaneous endoscopic ureterocalyceal anastomosis |
| KAH54 | Percutaneous endoscopic incision or dilatation of neck of calyx |
| KAH55 | Retrograde ureteronephroscopic incision or dilatation of neck of calyx |
| KAH61 | Percutaneous endoscopic incision of ureteropelvic junction |
| KAH62 | Endopyelotomy |
| KAH70 | Freeing of adhesions of ureteropelvic junction |
| KAH71 | Percutaneous endoscopic freeing of adhesions of ureteropelvic junction |
| KAH80 | Nephropexy |
| KAH81 | Percutaneous endoscopic nephropexy |
| KAH96 | Other reconstruction of kidney or pelvis of kidney |
| KAH97 | Other percutaneous endoscopic reconstruction of kidney or pelvis of kidney |
| KAH98 | Other transluminal endoscopic reconstruction of kidney or pelvis of kidney |
|  | **External drainage of pyelum** |
| KAJ00 | Nephropyelostomy, open |
| KAJ01 | Percutaneous nephropyelostomy |
| KAJ02 | Retrograde ureteronephroscopic nephrostomy |
| KAJ96 | Other external drainage of pelvis of kidney |
| KAJ97 | Other percutaneous endoscopic external drainage of pelvis of kidney |
| KAJ98 | Other transluminal endoscopic external drainage of pelvis of kidney |
|  | **Other surgery** |
| KAW96 | Other operation on kidney or pelvis of kidney |
| KAW97 | Other percutaneous endoscopic operation on kidney or pelvis of kidney |
| KAW98 | Other transluminal endoscopic operation on kidney or pelvis of kidney |
|  | **Exploration of the ureter** |
| KBA00 | Exploration of ureter |
| KBA01 | Percutaneous nephroureteroscopy |
| KBA10 | Exploratory ureterotomy |
| KBA11 | Percutaneous endoscopic exploratory ureterotomy |
| KBA96 | Other exploration of ureter |
| KBA97 | Other percutaneous endoscopy or endoscopic incision of ureter |
|  | **Excision/resection of the ureter** |
| KBC00 | Ureterectomy |
| KBC01 | Percutaneous endoscopic ureterectomy |
| KBD01 | Percutaneous endoscopic partial excision of ureter |
| KBD20 | Destruction of tumour of ureter |
| KBD21 | Percutaneous endoscopic destruction of tumour of ureter |
| KBD22 | Retrograde ureteroscopic destruction of tumour of ureter |
| KBD30 | Excision of stump of ureter |
| KBD31 | Percutaneous endoscopic excision of stump of ureter |
| KBD96 | Other partial excision of ureter or destruction of tumour of ureter |
| KBD97 | Other percutaneous endoscopic partial excision of ureter or destruction of tumour of ureter |
|  | **Ureter stone removal** |
| KBE00 | Ureterolithotomy |
| KBE01 | Percutaneous endoscopic ureterolithotomy |
| KBE12 | Transluminal endoscopic extraction of calculus of ureter |
| KBE22 | Transluminal endoscopic dislodgement of impacted calculus from ureter |
| KBE96 | Other operation for calculus of ureter |
| KBE97 | Other percutaneous endoscopic operation for calculus of ureter |
| KBE98 | Other transluminal endoscopic operation for calculus of ureter |
|  | **Foreign body removal from ureter** |
| KBF00 | Removal of foreign body from ureter |
| KBF01 | Percutaneous endoscopic removal of foreign body from ureter |
| KBF02 | Transluminal removal of foreign body from ureter |
|  | **Reconstruction and anastomosis of ureter** |
| KBH00 | Suture of ureter |
| KBH01 | Percutaneous endoscopic suture of ureter |
| KBH06 | Ureteroureterostomy |
| KBH20 | Replantation of ureter |
| KBH21 | Percutaneous endoscopic replantation of ureter |
| KBH30 | Ileal replacement of ureter |
| KBH40 | Plastic repair of ureter |
| KBH45 | Endoureterotomy |
| KBH50 | Ureterolysis |
| KBH62 | Transluminal dilatation of ureter |
| KBH96 | Other repair or connection of ureter |
| KBH97 | Other percutaneous endoscopic repair or connection of ureter |
| KBH98 | Other transluminal endoscopic repair of ureter |
|  | **Percutaneous drainage of ureter** |
| KBJ00 | Cutaneous ureterostomy |
| KBJ01 | Percutaneous endoscopic cutaneous ureterostomy |
| KBJ10 | Cutaneous ureteroenterostomy |
| KBJ11 | Endoscopic cutaneous ureteroenterostomy |
| KBJ20 | Cutaneous ureteroenterostomy with reservoir |
| KBJ40 | Ureteroenterostomy |
| KBJ60 | Anastomosis of ureter to urethra with interposition of ileum |
| KBJ70 | Removal of calculus from ileal conduit or reservoir |
| KBJ72 | Transluminal endoscopic removal of calculus from ileal conduit or reservoir |
| KBJ80 | Operation for malfunction of urinary diversion |
| KBJ96 | Other urinary diversion from ureter or related operation |
| KBJ97 | Other percutaneous endoscopic urinary diversion from ureter or related operation |
| KBJ98 | Other transluminal endoscopic procedure relating to urinary diversion from ureter |
|  | **Ureter stents** |
| KBV00 | Insertion of stent into ureter |
| KBV01 | Nephroscopic insertion of stent into ureter |
| KBV02 | Transluminal insertion of stent into ureter |
| KBV05 | Cystoscopic repositioning of stent in ureter |
| KBV10 | Removal of stent from ureter |
| KBV11 | Nephroscopic removal of stent from ureter |
| KBV12 | Transluminal endoscopic removal of stent from ureter |
| KBV15 | Cystoscopic change of stent in ureter |
| KBV22 | Cystoscopic dilatation of ureteric orifice |
| KBV32 | Cystoscopic incision of ureteric orifice |
| KBV40 | Incision or excision of ureterocele |
| KBV42 | Cystoscopic incision or excision of ureterocele |
| KBV52 | Cystoscopic injection therapy for vesicoureteral reflux |
|  | **Other ureter surgery** |
| KBW96 | Other operation on ureter |
| KBW97 | Other percutaneous endoscopic operation on ureter |
| KBW98 | Other transluminal endoscopic operation on ureter |
|  | **Vesical surgery inclusive reconstruction** |
| KCA00 | Exploratory cystotomy |
| KCA01 | Percutaneous endoscopic exploratory cystotomy |
| KCC00 | Cystectomy |
| KCC10 | Cystoprostatectomy |
| KCC11 | Cysto-prostato-vesiculectomia laparoscopica |
| KCC21 | Laparoscopic cystoprostatourethrectomy |
| KCC30 | Cystectomy with excision of female internal genital organs |
| KCC31 | Laparoscopic cystectomy with excision of female internal genital organs |
| KCC96 | Other cystectomy |
| KCC97 | Other laparoscopic cystectomy |
| KCD02 | Transurethral resection of bladder |
| KCD10 | Partial cystectomy |
| KCD11 | Percutaneous endoscopic partial cystectomy |
| KCD20 | Excision of diverticulum of bladder |
| KCD21 | Percutaneous endoscopic excision of diverticulum of bladder |
| KCD30 | Destruction of tumour of bladder |
| KCD32 | Cystoscopic destruction of tumour of bladder |
| KCD40 | Excision of urachus or other vesicocutaneous fistula |
| KCD96 | Other partial excision or destruction of tumour of bladder |
| KCD97 | Other percutaneous endoscopic partial excision of bladder or destruction of tumour of bladder |
| KCD98 | Other cystoscopic partial excision of bladder or destruction of tumour of bladder |
| KCH00 | Suture of bladder |
| KCH01 | Percutaneous endoscopic suture of bladder |
| KCH10 | Enterocystoplasty |
| KCH11 | Percutaneous endoscopic enterocystoplasty |
| KCH20 | Reduction cystoplasty |
| KCH21 | Percutaneous endoscopic reduction cystoplasty |
| KCH30 | Closure of vesicointestinal fistula |
| KCH40 | Incision or resection of bladder neck |
| KCH42 | Transurethral incision or resection of bladder neck |
| KCH96 | Other reconstructive operation on bladder |
| KCH97 | Other percutaneous endoscopic reconstructive operation on bladder |
| KCH98 | Other transluminal endoscopic reconstructive operation on bladder |
| KCJ00 | Cystostomy |
| KCJ10 | Cutaneous cystoenterostomy |
| KCJ20 | Continent cutaneous cystoenterostomy |
| KCJ96 | Other cystostomy |
| KCV02 | Cystoscopic balloon distention of bladder |
| KCV10 | Denervation of bladder |
| KCV11 | Percutaneous endoscopic denervation of bladder |
| KCV20 | Freeing of bladder |
| KCV21 | Percutaneous endoscopic freeing of bladder |
|  | **Urtehral surgery** |
| KDC00 | Urethrectomy |
| KDD00 | Partial excision of urethra |
| KDD10 | Excision of diverticulum of urethra |
| KDD30 | Destruction of tumour of urethra |
| KDD32 | Urethroscopic destruction of tumour of urethra |
| KDD40 | Resection of external sphincter of urethra |
| KDD42 | Urethroscopic resection of external sphincter of urethra |
| KDD50 | Excision of urethral valve |
| KDD52 | Urethroscopic excision of urethral valve |
| KDD80 | Partial excision of urethra and repair using graft or flap |
| KDD96 | Other partial excision of urethra |
|  | **Surgery for incontinence** |
| KDG00 | Retropubic suspension of urethra |
| KDG01 | Percutaneous endoscopic retropubic suspension of urethra |
| KDG10 | Abdominovaginal suspension of bladder neck |
| KDG20 | Abdominal colposuspension |
| KDG21 | Percutaneous endoscopic colposuspension |
| KDG30 | Suprapubic sling urethrocystopexy |
| KDG31 | Percutaneous endoscopic suprapubic sling urethrocystopexy |
| KDG40 | Suprapubic urethrocystopexy |
| KDG41 | Percutaneous endoscopic suprapubic urethrocystopexy |
| KDG43 | Transobturatorial sling urethrocystopexy |
| KDG50 | Transabdominal plastic repair of pelvic floor for urinary incontinence |
| KDG60 | Implantation of adjustable expander around bladder neck |
| KDG70 | Exploration of urethra |
| KDG96 | Other operation on urethra or bladder neck for incontinence |
| KDG97 | Other percutaneous endoscopic operation on urethra or bladder neck for incontinence |
|  | **Reconstruction of urethra** |
| KDH00 | Suture of urethra |
| KDH10 | Meatoplasty of urethra |
| KDH30 | Closure of urethrocutaneous fistula |
| KDH50 | Closure of urethrointestinal fistula |
| KDH62 | Urethroscopic recanalisation of urethra |
| KDH70 | Plastic repair of stricture of urethra |
| KDH96 | Other reconstructive operation on urethra |
| KDH98 | Other transluminal endoscopic reconstructive operation on urethra |
|  | **Sphincter prosthesis** |
| KDK00 | Implantation of artificial urinary sphincter around bladder neck |
| KDK01 | Laparoscopic implantation of artificial sphincter around bladder neck |
| KDK10 | Implantation of artificial urinary sphincter around bulbar urethra |
| KDK30 | Revision of artificial urethral sphincter |
| KDK40 | Removal of artificial urethral sphincter |
|  | **Prostata surgery** |
| KEA00 | Exploration of prostate |
| KEA10 | Prostatotomy |
| KEA20 | Incision of seminal vesicle |
| KEC00 | Retropubic radical prostatectomy |
| KEC01 | Percutaneous endoscopic radical prostatectomy |
| KEC10 | Perineal radical prostatectomy |
| KEC20 | Transsacral radical prostatectomy |
| KED00 | Transvesical prostatectomy |
| KED22 | Transurethral resection of prostate |
| KED32 | Transurethral incision of prostate |
| KEW96 | Other operation on prostate or seminal vesicle |
| KEW97 | Other percutaneous endoscopic operation on prostate or seminal vesicles |
|  | **Retroperitoneal surgery** |
| KKA00 | Exploration of retroperitoneal space |
| KKA01 | Percutaneous exploration of retroperitoneal space |
| KKA10 | Biopsy of retroperitoneal tissue |
| KKA20 | Incision of retroperitoneal space |
| KKA21 | Percutaneous endoscopic drainage of retroperitoneal space |
| KKB10 | Excision of retroperitoneal tumour |
| KKB11 | Percutaneous endoscopic excision of retroperitoneal tumour |
| KKB20 | Excision of retroperitoneal fistula |
| KKB21 | Percutaneous endoscopic excision of retroperitoneal fistula |
| KKF00 | Removal of retroperitoneal foreign body |
| KKF01 | Percutaneous endoscopic removal of retroperitoneal foreign body |
| KKW96 | Other operation on retroperitoneal space |
| KKW97 | Other percutaneous endoscopic operation on retroperitoneal space |
| KXK00 | Intralesional excision of extensive tumour of retroperitoneal space without defined origin in chapter K two-character group |
| KXK03 | Marginal excision of extensive tumour of retroperitoneal space without defined origin in chapter K two-character group |
| KXK06 | Wide excision of extensive tumour of retroperitoneal space without defined origin in chapter K two-character group |
|  | **Ovarial surgery** |
| LAA00 | Puncture of ovarian cyst |
| LAA01 | Laparoscopic puncture of ovarian cyst |
| LAA11 | Laparoscopic recovery of oocyte |
| LAA31 | Ovarioscopy |
| LAA96 | Other puncture of ovary |
| LAA97 | Other laparoscopic puncture of ovary |
| LAB00 | Ovariotomy |
| LAB01 | Laparoscopic ovariotomy |
| LAB10 | Biopsy of ovary |
| LAC11 | Laparoscopic fenestration of ovarian cyst |
| LAC20 | Destruction of lesion of ovary |
| LAC21 | Laparoscopic destruction of lesion of ovary |
| LAC30 | Excision of paraovarian cyst |
| LAC31 | Laparoscopic excision of paraovarian cyst |
| LAC96 | Other excision or destruction of lesion of ovary |
| LAC97 | Other laparoscopic excision or destruction of lesion of ovary |
| LAD00 | Partial excision of ovary |
| LAD01 | Laparoscopic partial excision of ovary |
| LAE10 | Unilateral oophorectomy |
| LAE11 | Unilateral laparoscopic oophorectomy |
| LAE20 | Bilateral oophorectomy |
| LAE21 | Bilateral laparoscopic oophorectomy |
| LAF00 | Unilateral salpingo-oophorectomy |
| LAF01 | Laparoscopic unilateral salpingo-oophorectomy |
| LAF10 | Bilateral salpingo-oophorectomy |
| LAF11 | Laparoscopic bilateral salpingo-oophorectomy |
| LAF20 | Unilateral transvaginal salpingo-oophorectomy |
| LAF30 | Bilateral transvaginal salpingo-oophorectomy |
| LAG00 | Freeing of adhesions of ovary |
| LAG01 | Laparoscopic freeing of adhesions of ovary |
| LAG11 | Laparoscopic oophoropexy |
| LAG20 | Detorsion of ovary |
| LAG21 | Laparoscopic detorsion of ovary |
| LAG96 | Other reconstructive operation on ovary |
| LAG97 | Other laparoscopic reconstructive operation on ovary |
| LAW96 | Other operation on ovary |
| LAW97 | Other laparoscopic operation on ovary |
|  | **Tube surgery** |
| LBA01 | Laparoscopic puncture of Fallopian tube |
| LBA07 | Salpingoscopy |
| LBB00 | Biopsy of Fallopian tube |
| LBB01 | Laparoscopic biopsy of Fallopian tube |
| LBB04 | Salpingoscopic biopsy of Fallopian tube |
| LBB11 | Laparoscopic needle biopsy of Fallopian tube |
| LBC00 | Percutaneous injection into tubal pregnancy |
| LBC07 | Laparoscopic injection into tubal pregnancy |
| LBC08 | Falloposcopic injection into tubal pregnancy |
| LBC10 | Removal of products of conception from Fallopian tube |
| LBC11 | Laparoscopic removal of products of conception from Fallopian tube |
| LBC20 | Salpingotomy and removal of products of conception |
| LBC21 | Laparoscopic salpingotomy and removal of products of conception |
| LBC96 | Other tube conserving operation for tubal pregnancy |
| LBC97 | Other laparoscopic tube conserving operation for tubal pregnancy |
| LBC98 | Other transluminal endoscopic tube conserving operation for tubal pregnancy |
| LBD00 | Partial excision of Fallopian tube |
| LBD01 | Laparoscopic partial excision of Fallopian tube |
| LBE00 | Salpingectomy |
| LBE01 | Laparoscopic salpingectomy |
| LBF00 | Perfusion of Fallopian tube |
| LBF01 | Laparoscopic perfusion of Fallopian tube |
| LBF03 | Perfusion of Fallopian tube after reconstruction |
| LBF11 | Laparoscopy for assisted fertilisation |
| LBF20 | Transcervical catheter salpingoplasty |
| LBF30 | Salpingolysis |
| LBF40 | Fimbrioplasty |
| LBF41 | Laparoscopic fimbrioplasty |
| LBF50 | Salpingostomy |
| LBF51 | Laparoscopic salpingostomy |
| LBF60 | Partial excision and anastomosis of Fallopian tube |
| LBF61 | Laparoscopic partial excision and anastomosis of Fallopian tube |
| LBF70 | Partial excision and reimplantation of Fallopian tube |
| LBF96 | Other operation on Fallopian tube for infertility |
| LBF97 | Other laparoscopic operation on Fallopian tube for infertility |
|  | **Uterus surgery** |
| LCA00 | Biopsy of uterus or uterine ligaments |
| LCA01 | Laparoscopic biopsy of uterus or uterine ligaments |
| LCA06 | Biopsy of endometrium |
| LCA10 | Curettage of body of uterus |
| LCA13 | Curettage of cervix and body of uterus |
| LCA16 | Destruction of endometrium |
| LCA20 | Removal of foreign body from uterus |
| LCA22 | Hysteroscopic removal of foreign body |
| LCA30 | Transfer of oocyte or embryo to uterus in assisted fertilisation |
| LCA96 | Other intrauterine operation |
| LCA98 | Other transluminal endoscopic operation on uterus |
| LCB00 | Hysterotomy |
| LCB01 | Laparoscopic hysterotomy |
| LCB10 | Myomectomy |
| LCB11 | Laparoscopic myomectomy |
| LCB14 | Laparoscopic myolysis |
| LCB20 | Transvaginal myomectomy |
| LCB25 | Hysteroscopic excision of lesion |
| LCB28 | Hysteroscopic excision of endometrium |
| LCB32 | Hysteroscopic destruction of endometrium |
| LCB96 | Other excision of lesion of uterus |
| LCB97 | Other laparoscopic excision of lesion of uterus |
| LCB98 | Other transluminal endoscopic excision of lesion of uterus |
| LCC00 | Partial excision of uterus |
| LCC01 | Laparoscopic partial excision of uterus |
| LCC05 | Hysteroscopic excision of uterine wall |
| LCC10 | Supravaginal hysterectomy |
| LCC11 | Laparoscopic subtotal hysterectomy |
| LCC20 | Vaginal supravaginal hysterectomy |
| LCC96 | Other partial excision of uterus |
| LCC97 | Other laparoscopic partial excision of uterus |
| LCD00 | Hysterectomy |
| LCD01 | Total laparoscopic hysterectomy |
| LCD04 | Laparoscopic hysterectomy |
| LCD10 | Vaginal hysterectomy |
| LCD11 | Laparoscopically assisted vaginal hysterectomy |
| LCD30 | Radical hysterectomy |
| LCD31 | Radical laparoscopic hysterectomy |
| LCD40 | Radical vaginal hysterectomy |
| LCD96 | Other hysterectomy |
| LCD97 | Other laparoscopic hysterectomy |
| LCE00 | Anterior exenteration of female pelvis |
| LCE10 | Posterior exenteration of female pelvis |
| LCE20 | Total exenteration of female pelvis |
| LCE96 | Other exenteration of female pelvis |
| LCF00 | Excision of lesion of parametrium |
| LCF01 | Laparoscopic excision of lesion of parametrium |
| LCF10 | Excision of female varicocele |
| LCF11 | Laparoscopic excision of female varicocele |
| LCF96 | Other excision of lesion of parametrium |
| LCF97 | Other laparoscopic excision of lesion of parametrium |
|  | **Reconstruction of uterus** |
| LCG02 | Hysteroscopic freeing of adhesions |
| LCG10 | Suture of uterus |
| LCG11 | Laparoscopic suture of uterus |
| LCG20 | Hysteropexy |
| LCG21 | Laparoscopic hysteropexy |
| LCG30 | Resection or transcision of sacrouterine ligaments |
| LCG31 | Laparoscopic resection or transcision of sacrouterine ligaments |
| LCG40 | Reconstruction of uterus |
| LCG41 | Laparoscopic reconstruction of uterus |
| LCG96 | Other reconstructive operation on uterus |
| LCG97 | Other laparoscopic reconstructive operation on uterus |
| LCG98 | Other transluminal endoscopic reconstructive operation on uterus |
| LCW96 | Other operation on uterus and uterine ligaments |
| LCW97 | Other laparoscopic operation on uterus and uterine ligaments |
| LCW98 | Other hysteroscopic operation on uterus |
|  | **Cervix surgery** |
| LDC00 | Conisation of cervix uteri using knife |
| LDC03 | Conisation of cervix uteri using diathermy or laser |
| LDC10 | Partial excision of cervix uteri |
| LDC96 | Other excision of cervix uteri |
| LDD00 | Suture of cervix uteri |
| LDD10 | Plastic repair of cervix uteri |
| LDD20 | Removal of cerclage of cervix uteri |
|  | **Vagina surgery** |
| LEC00 | Partial excision of vagina |
| LEC10 | Excision of vaginal septum |
| LEE20 | Closure of urovaginal fistula using graft or flap |
| LEE30 | Closure of intestinovaginal fistula using graft or flap |
| LEE40 | Construction of vagina |
| LEE96 | Other repair of vagina |
| LEF00 | Anterior colporrhaphy |
| LEF03 | Posterior colporrhaphy |
| LEF10 | Colpoperineoplasty |
| LEF13 | Colpoperineoplasty and vaginal hysterectomy |
| LEF16 | Fullstendig prolapsplastikk |
| LEF20 | Partial colpocleisis |
| LEF23 | Complete colpocleisis |
| LEF34 | Cervixamputasjon ved prolaps |
| LEF40 | Vaginal repair of enterocele |
| LEF53 | Vaginal colpopexy after previous hysterectomy |
| LEF96 | Other operation for prolapse of uterus or vaginal vault |
| LEF97 | Other laparoscopic operation for prolapse of uterus or vaginal vault |
|  | **Incontinence surgery** |
| LEG00 | Vaginal urethrocystorrhaphy |
| LEG10 | Vaginal urethrocystopexy |
| LEG13 | Vaginal transobturatorial urethropexy |
| LEG20 | Plastic repair of female pelvic floor with levator division |
| LEG96 | Other vaginal operation for incontinence |
| LEW96 | Other operation on vagina |
| LEW97 | Other laparoscopic operation on vagina |
|  | **Sterilisation** |
| LGA00 | Sterilisation by ligature of Fallopian tubes |
| LGA10 | Sterilisation by destruction or division of Fallopian tubes |
| LGA11 | Laparoscopic sterilisation by destruction or division of Fallopian tubes |
| LGA20 | Sterilisation by constriction of Fallopian tubes |
| LGA21 | Laparoscopic sterilisation by constriction of Fallopian tubes |
| LGA22 | Hysteroscopic sterilisation |
| LGA24 | Laparascopic sterilsation with tube removal |
| LGA96 | Other female sterilisation |
| LGA97 | Other laparoscopic female sterilisation |
|  | **Suprarenal/visceral surgery** |
| PCA40 | Exploration of renal artery |
| PCA99 | Exploration of other visceral artery |
| PCB20 | Ligature of coeliac trunk and branches |
| PCB30 | Ligature of superior mesenteric artery |
| PCB40 | Ligature of renal artery |
| PCB99 | Ligature of other visceral artery |
| PCC10 | Suture of suprarenal or juxtarenal abdominal aorta |
| PCC20 | Suture of coeliac trunk and branches |
| PCC30 | Suture of superior mesenteric artery |
| PCC40 | Suture of renal artery |
| PCC99 | Suture of other visceral artery |
|  | **Embolectomy of visceral arteries** |
| PCE30 | Thrombectomy or embolectomy of superior mesenteric artery |
| PCE40 | Thrombectomy or embolectomy of renal artery |
| PCE99 | Thrombectomy or embolectomy of other visceral artery |
| PCF20 | Thrombendarterectomy of coeliac trunk and branches |
| PCF30 | Thrombendarterectomy of superior mesenteric artery |
| PCF40 | Thrombendarterectomy of renal artery |
| PCF99 | Thrombendarterectomy of other visceral artery |
|  | **Aneurysm in suprarenal arteries** |
| PCG10 | Operation for aneurysm of supracoeliac or juxtarenal abdominal aorta |
| PCG20 | Operation for aneurysm of coeliac trunk and branches |
| PCG30 | Operation for aneurysm of superior mesenteric artery |
| PCG40 | Operation for aneurysm of renal artery |
| PCG99 | Operation for aneurysm of other visceral artery |
|  | **Suprarenal bypass** |
| PCH10 | Bypass from supracoeliac or juxtarenal abdominal aorta |
| PCH20 | Bypass to/from coeliac trunk and branches |
| PCH30 | Bypass to/from superior mesenteric artery |
| PCH40 | Bypass to/from renal artery |
| PCH99 | Bypass from other visceral artery |
| PCJ30 | Transposition of superior mesenteric artery |
| PCJ40 | Transposition of renal artery |
| PCJ99 | Transposition of other visceral artery |
|  | **Visceral reimplantation** |
| PCK20 | Reimplantation of coeliac trunk and branches |
| PCK30 | Reimplantation of superior mesenteric artery |
| PCK40 | Reimplantation of renal artery |
| PCK50 | Reimplantation of inferior mesenteric artery |
| PCK99 | Reimplantation of other visceral artery |
| PCN20 | Plastic repair of coeliac trunk and branches |
| PCN30 | Plastic repair of superior mesenteric artery |
| PCN40 | Plastic repair of renal artery |
| PCN99 | Plastic repair of other visceral artery |
| PCS40 | Endoscopic operation on renal artery |
| PCS99 | Endoscopic operation on other visceral artery |
| PCW99 | Other operation on suprarenal abdominal aorta and visceral arteries |
|  | **Infrarenal abdominal aorta surgery** |
| PDA10 | Exploration of infrarenal abdominal aorta |
| PDA30 | Exploration of iliac artery |
| PDC10 | Suture of infrarenal abdominal aorta |
| PDC30 | Suture of iliac artery |
| PDE10 | Thrombectomy or embolectomy of infrarenal abdominal aorta |
| PDE30 | Thrombectomy or embolectomy of iliac artery |
| PDF10 | Thrombendarterectomy of infrarenal abdominal aorta |
| PDF30 | Thrombendarterectomy of iliac artery |
| PDG10 | Operation on infrarenal abdominal aorta for aneurysm |
| PDG20 | Bypass from aorta to iliac artery for aneurysm |
| PDG30 | Operation on iliac artery for aneurysm |
| PDG35 | Bypass from iliac to femoral artery for aneurysm |
| PDG99 | Other operation for aneurysm of infrarenal abdominal aorta and iliac arteries |
|  | **Bypass aortoiliacal** |
| PDH10 | Bypass from infrarenal abdominal aorta |
| PDH20 | Bypass from aorta to iliac artery |
| PDH21 | Bypass from aorta to bilateral iliac arteries |
| PDH22 | Bypass from aorta to iliac and contralateral femoral artery |
| PDH23 | Bypass from aorta to femoral artery |
| PDH24 | Bypass from aorta to bilateral femoral arteries |
| PDH30 | Bypass from iliac artery |
| PDH35 | Bypass from iliac to femoral artery |
| PDH99 | Other bypass from abdominal aorta or iliac artery |
|  | **Major vessel plasty** |
| PDN10 | Plastic repair of infrarenal abdominal aorta |
| PDN30 | Plastic repair of iliac artery |
| PDS10 | Endoscopic operation on infrarenal abdominal aorta |
| PDS30 | Endoscopic operation on iliac artery |
| PDW99 | Other operation on infrarenal abdominal aorta and iliac arteries and distal connections |
|  | **Femoral artery surgery** |
| PEA10 | Exploration of femoral artery |
| PEA11 | Exploration of deep femoral artery |
| PEA12 | Exploration of superficial femoral artery |
| PEC10 | Suture of femoral artery |
| PEC11 | Suture of deep femoral artery |
| PEC12 | Suture of superficial femoral artery |
| PEE10 | Thrombectomy or embolectomy of femoral artery |
| PEE11 | Thrombectomy or embolectomy of deep femoral artery |
| PEE12 | Thrombectomy or embolectomy of superficial femoral artery |
| PEF10 | Thrombendarterectomy of femoral artery |
| PEF11 | Thrombendarterectomy of deep femoral artery |
| PEF12 | Thrombendarterectomy of superficial femoral artery |
| PEH10 | Bypass from femoral artery |
| PEH11 | Bypass from deep femoral artery |
| PEH12 | Bypass from superficial femoral artery |
| PEH20 | Bypass from femoral to popliteal artery above knee |
| PEH30 | Bypass from femoral artery to popliteal artery below knee |
| PEL10 | Construction of arteriovenous fistula from femoral artery |
| PEN10 | Plastic repair of femoral artery |
| PEN11 | Plastic repair of deep femoral artery |
| PEN12 | Plastic repair of superficial femoral artery |
| PES10 | Endoscopic operation on femoral artery |
| PES11 | Endoscopic operation on deep femoral artery |
| PES12 | Endoscopic operation on superficial femoral artery |
| PEW99 | Other operation on femoral artery with branches and connection to popliteal artery |
